# Fast Reverse Design of 4D‐Printed Voxelized Composite Structures Using Deep Learning and Evolutionary Algorithm

**DOI:** 10.1002/advs.202407825

**Published:** 2025-02-01

**Authors:** Mengtao Wang, Zaiyang Liu, Hidemitsu Furukawa, Zhuo Li, Yifei Ge, Yifan Xu, Zhe Qiu, Yang Tian, Zhongkui Wang, Ren Xu, Lin Meng

**Affiliations:** ^1^ Department of Electronic and Computer Engineering Ritsumeikan University Shiga 525‐8577 Japan; ^2^ Department of Robotics Ritsumeikan University Shiga 525‐8577 Japan; ^3^ Department of Mechanical Systems Engineering Yamagata University Yonezawa 992‐8510 Japan; ^4^ School of Medicine Xiamen University Xiamen Fujian 361100 China

**Keywords:** 4D printing, deep learning, evolutionary algorithm, hydrogel, voxel assembly

## Abstract

Designing voxelized composite structures via 4D printing involves creating voxel units with distinct material properties that transform in response to stimuli; however, optimally distributing these properties to achieve specific target shapes remains a significant challenge. This study introduces an optimization method combining deep learning (DL) and an evolutionary algorithm, focusing on a solvent‐responsive hydrogel as the target material. A sequence‐enhanced parallel convolutional neural network is developed and generated a dataset through finite element simulations. This DL model enables high‐precision prediction of hydrogel deformation. Furthermore, a progressive evolutionary algorithm (PEA) is proposed by integrating the DL model to construct a DL‐PEA framework. This framework supports rapid reverse engineering of the desired shape, and the average design time for specified target shapes is reduced to ≈3.04 s. The present findings illustrate how 4D printing of optimized hydrogel designs can effectively transform in response to environmental stimuli. This work provides a new perspective on the application of hydrogels in 4D printing and presents an efficient tool for optimizing 4D‐printed voxelized composite structures.

## Graphical abstract

A DL‐PEA system is developed by combining deep learning and evolutionary algorithms for the fast inverse design of 4D‐printed voxelized composite structures. DL‐PEA can accurately achieve the inverse design of target shapes by applying voxelization to hydrogels. This study provides an efficient tool for optimizing 4D‐printed voxelized composite structures.

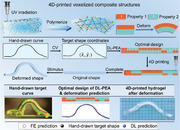

## 1 Introduction

Building upon 3D printing, 4D printing technology introduces time as the fourth dimension through the use of special stimulus‐responsive materials such as shape memory polymers (SMPs),^[^ ^1^, ^2^ ^]^ hydrogels,^[^ ^3^, ^4^, ^5^ ^]^ and liquid crystal elastomers (LCEs).^[^ ^6^, ^7^, ^8^, ^9^ ^]^ These materials enable printed structures to alter their shape or properties in response to external stimuli (e.g., magnetic fields, light, and temperature). Such capabilities significantly enhance the flexibility in design and dynamic adaptability of products, facilitating the creation of complex, multifunctional intelligent structures. In recent years, the potential applications of 4D printing technology have expanded significantly across various fields, including biomedical,^[^ ^10^, ^11^ ^]^ smart textiles,^[^ ^12^, ^13^ ^]^ and soft robotics,^[^ ^14^, ^15^ ^]^ thereby driving advancements in materials science and manufacturing technologies.

**Figure 1:**
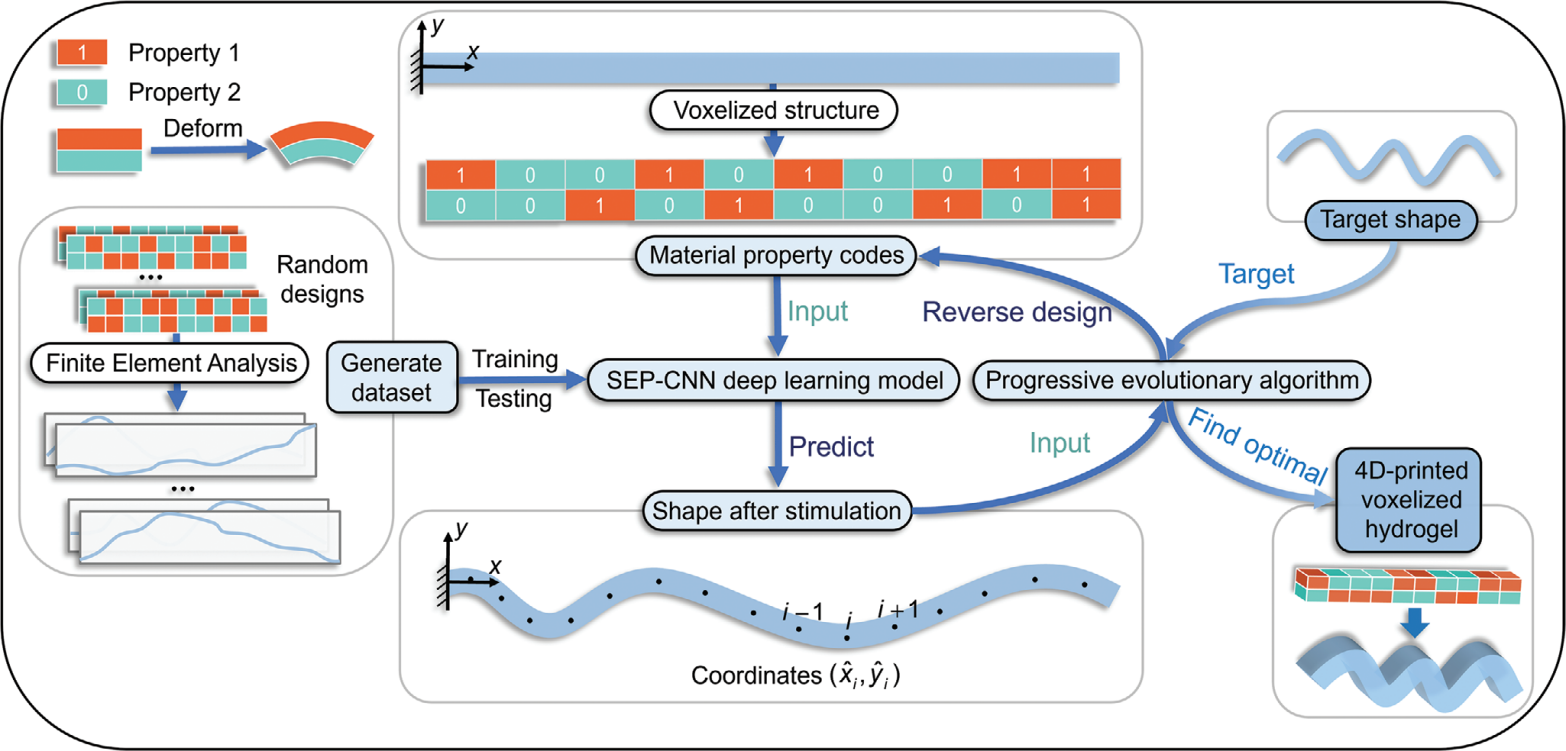
Schematic of fast reverse design of 4D‐printed voxelized composite structures. A solvent‐responsive hydrogel exhibits varying expansion rates and serves as a carrier, where “1” denotes an active material property with a larger expansion rate, and “0” indicates a passive material property with a smaller expansion rate. These hydrogels are voxelized and digitally encoded, and their deformation dataset is generated through FE simulation. This dataset is then used to train and test the SEP‐CNN model, which predicts the deformation of arbitrarily encoded voxelized composite structures. Progressive evolutionary algorithm (PEA) is employed to facilitate the reverse design for the targeted shape changes. The integration of DL and PEA ensures rapid identification of the optimal material distribution for the desired shape.

Active composites, which comprise two or more materials with differing stimulus‐responsive properties, represent a novel category of functional materials.^[^ ^16^ ^]^ By voxelizing these composite structures and assigning distinct material properties to each voxel unit, theoretically unlimited design possibilities can be created. In practice, each voxel unit within a voxelized composite typically consists of the same material type, but these materials may vary in specific properties (e.g., expansion, stiffness, and modulus of elasticity). These variations enable the material to respond differently to environmental stimuli, thereby altering its shape and properties. The design flexibility of such structures can be greatly enhanced by integrating voxelized composite materials with 4D printing technology. This integration utilizes the precise control of 4D printing to assign distinct properties to each voxel, enhancing the functionality of the material.^[^ ^17^, ^18^ ^]^ For instance, consider a voxelized composite structure featuring two different material properties, as illustrated in **Figure** **1**, which depicts a bilayer structure comprising two voxel units that deform in response to environmental stimuli. By segmenting a long, beam‐shaped structure into numerous voxel units, a complex spatial arrangement of two material properties is achieved. The spatial distribution of materials critically influences the deformation behavior of the structure. As the number of voxel units increases, the possibilities for designing spatial distributions expand exponentially. This expansion broadens the design space to satisfy a wider array of requirements. However, designing the optimal material distribution to achieve the desired shape change poses a significant challenge. Given the vast and complex array of design options, relying solely on empirical judgment or extensive experimental searches to ensure optimal design is impractical. Such methods are not only time‐consuming but also resource‐intensive.

Leveraging the advanced computational capabilities of modern computers, modeling and simulation technologies enable the visualization of material shape changes or performance under specific parameters, effectively addressing the time‐intensive and resource‐consuming nature of physical experiments. For instance, the finite element (FE) method is a well‐established technique and extensively utilized for the predictive analysis of active composite materials.^[^ ^19^, ^20^, ^21^ ^]^ However, the FE method can be time‐consuming when applied to more complex structures, which restricts its use in large‐scale structural predictions. In recent years, the rapid advancements in deep learning (DL) have provided new opportunities for fast and accurate predictions. As a data‐driven approach, DL is particularly effective for analyzing the mechanical responses of complex materials or structures, including the deformation and structural vibration of composite materials,^[^ ^22^, ^23^, ^24^, ^25^ ^]^ the dynamic response of continuously deformable bodies,^[^ ^26^, ^27^, ^28^ ^]^ and the stress fields within composite materials,^[^ ^29^, ^30^, ^31^, ^32^ ^]^ among other problems.

The reverse design problem, which involves determining the optimal material distribution for a specified target shape, depends on accurate forward deformation prediction as well as on the integration of optimization algorithms. For example, various gradient‐based topology optimization methods,^[^ ^33^ ^]^ including solid isotropic material with penalization,^[^ ^34^ ^]^ level set methods,^[^ ^35^ ^]^ topological derivative methods,^[^ ^36^ ^]^ and method of moving asymptotes^[^ ^37^ ^]^ are crucial in addressing these challenges. However, these methods generally presume continuous or homogeneous material properties and may not be suitable for complex material structures that exhibit anisotropic or nonlinear characteristics. In such cases, voxelized composite materials, which are discretized structures composed of discrete voxel units, present a different set of challenges as they do not exhibit the characteristics of continuous variables. Evolutionary algorithms (EAs), which simulate the evolutionary processes of natural selection and heredity, are more appropriate for optimizing these structures.^[^ ^38^ ^]^ The integration of EAs with FE methods has shown considerable success in the reverse design of voxel‐based active composites.^[^ ^39^, ^40^ ^]^ However, this FE‐based EA approach requires numerous FE simulations, leading to lengthy computation times and high computational costs. DL can serve as a highly efficient alternative to traditional FE simulations, significantly reducing both computing time and cost.^[^ ^41^, ^42^ ^]^ Combining DL with EA facilitates a rapid and accurate reverse design process, offering a novel and efficient approach for the optimal design of voxelized composite materials.

In this study, we introduce an optimization strategy that combines DL and EA to efficiently and accurately predict and design the deformation behavior of voxelized composites. The focus is on a solvent‐responsive hydrogel that exhibits varying swelling rates and can be formed into voxel‐based beam structures using 4D printing technology. The entire optimization process is illustrated in Figure 1. We developed a sequence‐enhanced parallel convolutional neural network (SEP‐CNN) DL model to predict the forward deformation of the material. The model is trained using a dataset generated from randomized FE simulations. The SEP‐CNN model leverages the parallel processing capabilities of convolutional neural networks (CNNs) and the sequential data handling of recurrent neural networks (RNNs), enabling highly efficient and accurate predictions of hydrogel deformation. For the reverse design of materials, we introduce a progressive evolutionary algorithm (PEA) that optimizes voxel units column‐by‐column, which exceeds the efficiency of traditional EA methods. When combined with the SEP‐CNN, the PEA can identify the optimal material distribution for achieving the desired target shape through up to five generations of evolution, with an average design time of only 3.04 s, demonstrating remarkable efficiency. In contrast, using PEA in conjunction with FE simulations for similar reverse design tasks requires ≈180 h, which is an impractical duration for mass production. This optimization method is applicable to complex geometries such as biological skeletal lines or hand‐drawn curves, which enabled rapid determination of optimal material distribution and laying the groundwork for future applications. Moreover, this efficient optimization strategy can be adapted to address the optimization challenges of various materials or structures, thus providing a valuable tool for optimizing 4D‐printed voxelized composite structures.

## 2 Results

### 2.1 4D Printing of Hydrogels

**Figure 2:**
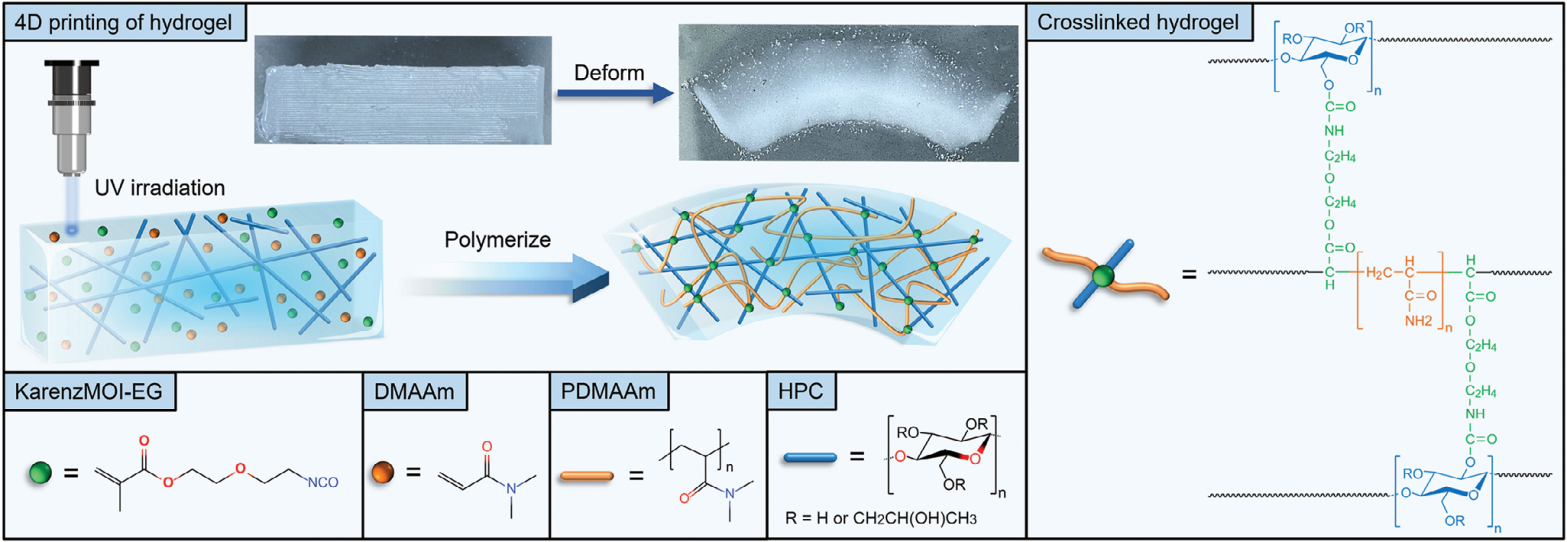
Schematic of hydrogel 4D printing. Gel solutions consisting of various compounds polymerize into crosslinked hydrogels by self‐reaction after UV irradiation. Hydrogel exhibits a higher crosslink density on the side exposed to UV light and a lower crosslink density on the other side. Two sides of the hydrogel exhibit different expansion rates, and thus, it changes shape after expanding by absorbing water.

In this study, we employ a solvent‐responsive hydrogel that can achieve voxelized structures as a carrier to verify effectiveness of the proposed intelligent optimization method. **Figure** **2** illustrates the structural components of the hydrogel and a schematic of the developed 4D printing technology. The hydrogel is synthesized using the hydrophilic monomer N,N‐dimethylacrylamide (DMAAm), the polymer hydroxypropyl cellulose (HPC), and the crosslinker 2‐(2‐methacryloyloxyethyl)ethyl isocyanate (KarenzMOI‐EG). Additionally, the polymerization reaction is initiated using materials including the hydrophobic initiator TPO (diphenyl (2,4,6‐trimethylbenzoyl) phosphine oxide), the hydrophilic initiator TPO (lithium phenyl (2,4,6‐trimethylbenzoyl) phosphite), and the ultraviolet (UV) absorber KAYAPHOR AS150 (AS150). The gel solution containing the specified compounds is subjected to a light‐initiated polymerization reaction under UV irradiation, resulting in the formation of cross‐linked hydrogels. The degree of polymerization of the hydrogel is influenced by the intensity, speed, and number of UV scans. In 4D printing of hydrogels, structures with varying crosslinking densities are produced by precisely controlling the polymerization degree in each region, thereby achieving structures with gradient swelling properties. In this study, the intensity and speed of UV scans are maintained constant, while the number of UV scans is varied to print hydrogels with swelling gradients. Upon immersion in water, the printed hydrogel swells by absorbing water to produce a shape change. Regions with higher swelling rates bend toward regions with lower swelling rates, as demonstrated in the printed sample shown in Figure 2.

### 2.2 SEP‐CNN Model for Deformation Prediction

To acquire the coordinate data of a voxelized hydrogel post‐deformation, we immobilize the left edge of the hydrogel in the y‐direction, positioning the center of this edge at the coordinate origin during FE simulation, as depicted in Figure 1. Consequently, the material property inputs exhibit sequence characteristics and the deformed coordinate outputs also exhibit sequence characteristics, thereby establishing a sequence‐to‐sequence mapping relationship. RNNs are uniquely effective for processing sequential data, such as text and time‐series data.^[^ ^43^, ^44^, ^45^, ^46^ ^]^ In recent years, RNNs have also been applied to predict the dynamic mechanical responses of various materials and structures.^[^ ^47^, ^48^, ^49^, ^50^, ^51^ ^]^ Despite their significant advantages in sequence information processing, RNNs rely on the hidden state from a previous time step to process current inputs, which hampers the ability to parallelize data processing and reduces training efficiency. Conversely, the architecture of CNNs is better suited for parallel computation.^[^ ^52^, ^53^ ^]^ The effectiveness of CNNs in accurately predicting structural deformations has also been confirmed.^[^ ^54^, ^55^, ^56^ ^]^ Therefore, by combining the strengths of both CNNs and RNNs, we develop an SEP‐CNN model aimed at achieving high‐efficiency and high‐accuracy predictions of hydrogel deformation.

**Figure 3:**
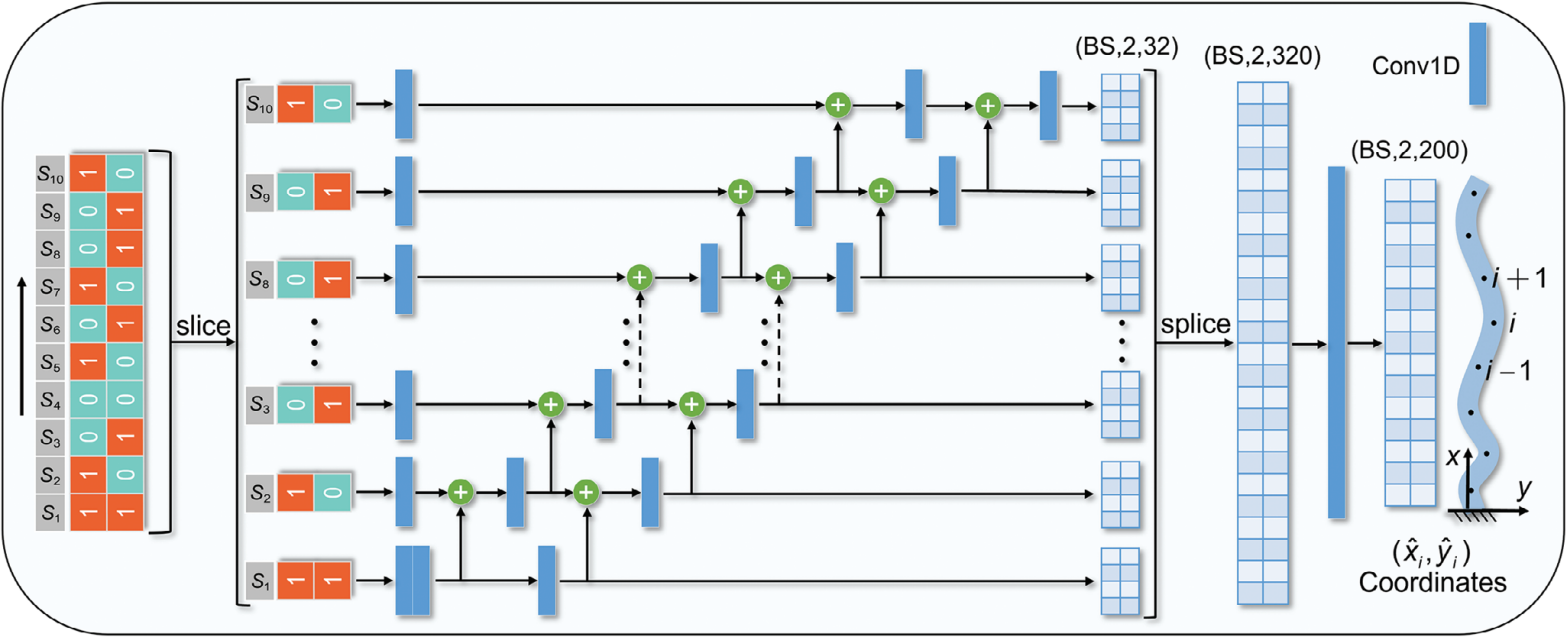
Schematic of SEP‐CNN architecture. The model inputs are material property encodings of voxelized hydrogel, which exhibit sequential characteristics in space. These encodings are sliced and fed sequentially into the corresponding Conv1D channels. The output from each channel is influenced by the inputs of the current channel as well as that of the previous channel. The model outputs the coordinate data of deformed hydrogels.

The network architecture of SEP‐CNN is depicted in **Figure** **3**. The inputs to the network are the material property encodings of voxelized hydrogel. We segment adjacent voxel units in the horizontal direction (y‐direction) according to their spatial order, denoted as the sequence $S=\{S_{1},\text{…},S_{i-1},S_{i},S_{i+1},\text{…},S_{n}\}$, and each *S*_*i*_ contains two random 0,1 encodings. The sequence S is processed sequentially, with each *S*_*i*_ fed in parallel to the corresponding channel equipped with 1D convolutional layers (Conv1D) that execute convolution operations. The term “parallel” indicates that the data in each channel can be processed simultaneously, unlike in RNNs where the computation at each time step must await completion of the previous step. The output of the model is the set of hydrogel deformation coordinates $\hat{P}=\left\{{\hat{P}}_{1},\text{…},{\hat{P}}_{i-1},{\hat{P}}_{i},{\hat{P}}_{i+1},\text{…},{\hat{P}}_{N}\right\}$, where each ${\hat{P}}_{i}$ represents a predicted coordinate $\left({\hat{x}}_{i},{\hat{y}}_{i}\right)$. Here, *N* = 20*n* signifies that each input *S*_*i*_ corresponds to 20 predicted coordinates. The objective of training this deep learning model is to minimize the discrepancy between the predicted values $\left({\hat{x}}_{i},{\hat{y}}_{i}\right)$ and the actual values (*x*_*i*_, *y*_*i*_), thereby achieving accurate predictions of hydrogel deformation.

In this type of prediction task, the mean square error (MSE) is commonly employed as the loss function for training models. MSE measures the discrepancy between the predicted values and the actual values. To further improve the prediction accuracy, we introduce the concepts of adjacent distance squared error (ADSE) and angle cosine squared error (ACSE), combined with MSE to form the *L*_*total*_ loss function. Where ADSE denotes the distance squared error between two neighboring points of the true and predicted values, and ACSE denotes the angle cosine squared error consisting of three neighboring points of the true and predicted values. The specific formulas and detailed explanations are described in the Experimental Section.

### 2.3 Prediction Performance of SEP‐CNN Model

**Figure 4:**
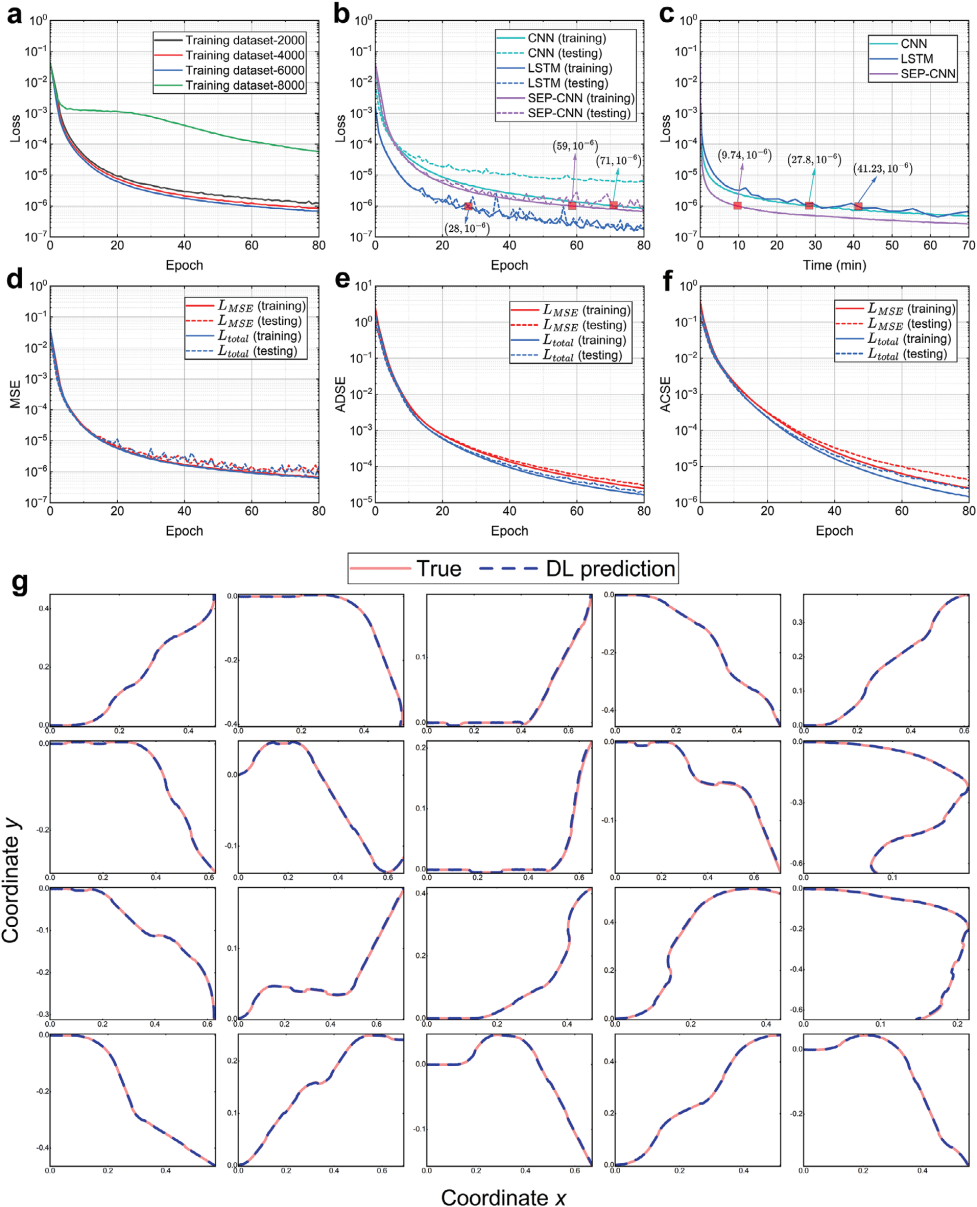
Performance of DL model deformation prediction. a) Sensitivity of the SEP‐CNN model to datasets of different sizes, with optimal performance on the dataset with 6000 samples. b) Variation of loss with training epoch for three DL models (LSTM, CNN, and SEP‐CNN), SEP‐CNN outperforms CNN, and LSTM performs best. c) The variations in training loss over time for the three models are analyzed, revealing that the SEP‐CNN model surpassed the LSTM model in terms of training efficiency. d) The *L*_*MSE*_ and *L*_*total*_ are used as loss functions for the SEP‐CNN model, respectively, comparing the variation of the computed MSE with the training epoch. e) The *L*_*MSE*_ and *L*_*total*_ are used as loss functions for the SEP‐CNN model, respectively, comparing the variation of the computed ADSE with the training epoch. f) The *L*_*MSE*_ and *L*_*total*_ are utilized as loss functions for the SEP‐CNN model, respectively, comparing the variation of the computed ACSE with the training epoch. g) In 20 randomly selected prediction examples, the predicted and true curves are highly coincident.

To select the optimal dataset for training a model to predict hydrogel shape changes with high precision, we evaluate model performance using training datasets of varying sizes (2000, 4000, 6000, and 8000 samples). The results in **Figure** **4**a indicate that the model achieves the best performance with a training dataset of 6000 samples, exhibiting lower loss values per epoch compared to other dataset sizes.

Figure 4b presents a performance comparison between SEP‐CNN, traditional CNN, and long short‐term memory (LSTM). All three models employ a 4‐layer network architecture (Figure S2, Supporting Information) with identical parameter settings. The results indicate that SEP‐CNN consistently exhibits lower training and testing losses per training epoch compared to traditional CNN. Additionally, SEP‐CNN maintains comparable performance on both the training and testing datasets, demonstrating its robust generalization capability. In contrast, the traditional CNN exhibits diminished performance on the testing dataset relative to the training dataset, and overfitting has occurred. When compared to LSTM, SEP‐CNN slightly underperforms in each training epoch, indicating that LSTM possesses superior learning capabilities for sequential data. Figure 4c illustrates the performance of the three models over the training duration. The data reveal that SEP‐CNN achieves lower loss values than both CNN and LSTM within the same training period. If a high accuracy threshold, such as a loss value of 10^−6^, is set as the criterion for terminating training, the required times are as follows: CNN requires 27.8 min, LSTM presumes 41.23 min, and SEP‐CNN only requires 9.74 min to satisfy this condition. In terms of training epochs, CNN, LSTM, and SEP‐CNN require 71, 59, and 28 epochs, respectively. This indicates that SEP‐CNN can process more data batches simultaneously and exhibits higher learning efficiency. Consequently, SEP‐CNN surpasses LSTM in performance due to its faster training speed.

In addition to assessing the training accuracy and speed of the model, we evaluated the performance of three DL models and FE simulation in terms of prediction speed. The results are summarized in **Table** **1**. We randomly generated 2000 hydrogel voxel structure encodings and performed FE simulations for deformation prediction using Abaqus, which required ≈34.67 h. In contrast, the DL model predictions required less than 1 min, demonstrating ultrahigh efficiency. The traditional CNN and the SEP‐CNN had similar prediction times, each requiring less than 3 s. Notably, SEP‐CNN significantly reduces the number of network parameters through a parallel‐connected CNN architecture, which is central to its enhanced training speed compared to traditional CNN. This design also significantly reduces the complexity of the model, thereby effectively avoiding overfitting. Although the LSTM model exhibited fewer network parameters, its higher computational complexity results in longer training and prediction times, with the prediction time being ≈16 times longer than that of SEP‐CNN.

**Table 1:** Time consumption for deformation prediction of random 2000 voxel structures using DL models (LSTM, CNN, and SEP‐CNN) and FE simulation, “h” for hours and “s” for seconds. Three DL models exhibit the same network depth and data shape transformation processes. All the tests were performed on a high‐performance computer equipped with an Intel Core i9‐12900F CPU and an NVIDIA GeForce RTX 3090 GPU.

Methods	Network parameters	Time consumption
FE	—	34.67h
LSTM	85,800	40.94s
CNN	817,160	2.38s
SEP‐CNN	255,560	2.51s

To demonstrate that the proposed loss function *L*_*total*_ enhances prediction accuracy, we compared it with the commonly used *L*_*MSE*_ in our model simulations. The results are presented in Figure 4d,e,f. Figure 4d illustrates that the MSE for both *L*_*total*_ and *L*_*MSE*_ remains consistent across training epochs. The results in Figure 4e,f reveals that *L*_*total*_ effectively reduces both ADSE and ACSE, thereby achieving higher prediction accuracy. Additionally, 20 randomly selected predicted examples from the test results are displayed in Figure 4g, where the predicted hydrogel deformation trajectories closely align with the actual trajectories.

### 2.4 Framework Design of DL‐based Progressive Evolutionary Algorithm (DL‐PEA)

**Figure 5:**
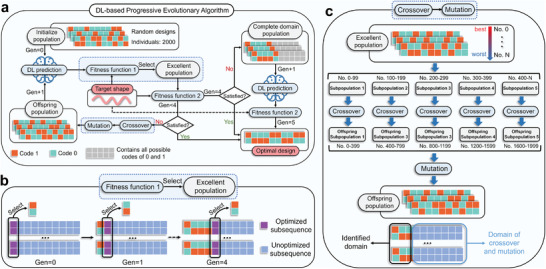
Flow diagram of the DL‐based PEA (DL‐PEA) approach for fast reverse design. a) Demonstration of the DL‐PEA Method: The DL model predicts deformation across all individuals in the population. Concurrently, the PEA optimizes the design of the target shape through evolutionary processes. b) Core of PEA: The process involves selecting superior populations by optimizing the local encoding on a column‐by‐column basis. c) Details of crossover and mutation: The superior population is ranked and segmented into five subpopulations, categorized from best to worst. Individuals within each subpopulation undergo crossover operations. Offspring populations are then produced through both crossover and mutation processes.

Thereafter, the DL model is integrated with the EA to facilitate rapid reverse design of the target shape. For the structural characteristics of voxelized hydrogels, we design a PEA that enables fast reverse design. As illustrated in **Figure** **5**a, the algorithm initiates by generating a random population of 2000 individuals. This population is processed by the well‐trained SEP‐CNN model, which predicts the deformed coordinate data for all individuals. The fitness of each individual is then assessed using the fitness function *f*_1_, which compares the predicted data with the target shape data. The fitness function *f*_1_ employs a progressive approach that processes the deformation coordinates column by column for each voxel unit. The specific definitions are provided in Equation (1).

(1) $$\begin{array}{c} f_{1}=\frac{1}{20}\sum_{i=20N_{gen}+1}^{20\left(N_{gen}+1\right)}\left[{\left(x_{i}-{\hat{x}}_{i}\right)}^{2}+{\left(y_{i}-{\hat{y}}_{i}\right)}^{2}\right] \end{array}$$

where *N*_*gen*_ denotes the generation (Gen) of population evolution. Each column of voxel units corresponds to 20 deformation coordinate points. The fitness function *f*_1_ computes the mean squared error (MSE) between the predicted and target coordinates for each column, based on the evolved *N*_*gen*_. A lower MSE value signifies higher fitness for an individual. Individuals are assessed using the fitness function *f*_1_, and those with higher fitness are selected as the superior population. This selection mechanism is depicted in Figure 5b. Given that the left edge of the hydrogel is fixed, the local deformation coordinates of each column voxel unit are influenced only by the preceding column and its own columns, not by subsequent columns. This structural characteristic allows for the determination of the optimal local solution on a column‐by‐column basis. During each evolutionary step, the superior population calculates the global fitness of each individual using the fitness function *f*_2_:

(2) $$\begin{array}{c} f_{2}=\frac{1}{N}\sum_{i=1}^{N}\left[{\left(x_{i}-{\hat{x}}_{i}\right)}^{2}-{\left(y_{i}-{\hat{y}}_{i}\right)}^{2}\right] \end{array}$$

The global fitness, denoted as *f*_2_, evaluates whether an individual meets predefined threshold conditions. When the generation count Gen < 4, and if *f*_2_ satisfies the required condition, the optimal coding design scheme is outputted. If the condition is not satisfied, the population undergoes crossover and mutation operations to generate offspring populations, which then continue the cyclic process of the PEA. At Gen = 4, the optimal encodings for the first five columns are determined, whereas those for the last five columns remain unresolved. Given that the last five columns collectively have 2^10^ = 1024 possible encoding combinations, it is feasible to traverse all possible encodings and evaluate each by calculating the global fitness *f*_2_. The individual with the highest fitness is selected as the optimal design solution. In conclusion, the PEA can identify the optimal design solution within a maximum of five evolutionary iterations.

Details of the crossover and mutation operations are illustrated in Figure 5c. Initially, the populations are organized and sequentially numbered based on their global fitness. Subsequently, the population is segmented into five subpopulations according to the coding sequence. The first four subpopulations comprise 100 individuals each, whereas the final subpopulation includes all remaining individuals. Each subpopulation then undergoes a crossover operation, resulting in progeny subpopulations each containing 400 individuals. Finally, all progeny subpopulations are subjected to mutations to form the final offspring populations. Note that both crossover and mutation processes are confined to unspecified domains. Further details on these operations are provided in Figures S4 and S5 (Supporting Information).

### 2.5 Performance of DL‐PEA for Fast Reverse Design

**Figure 6:**
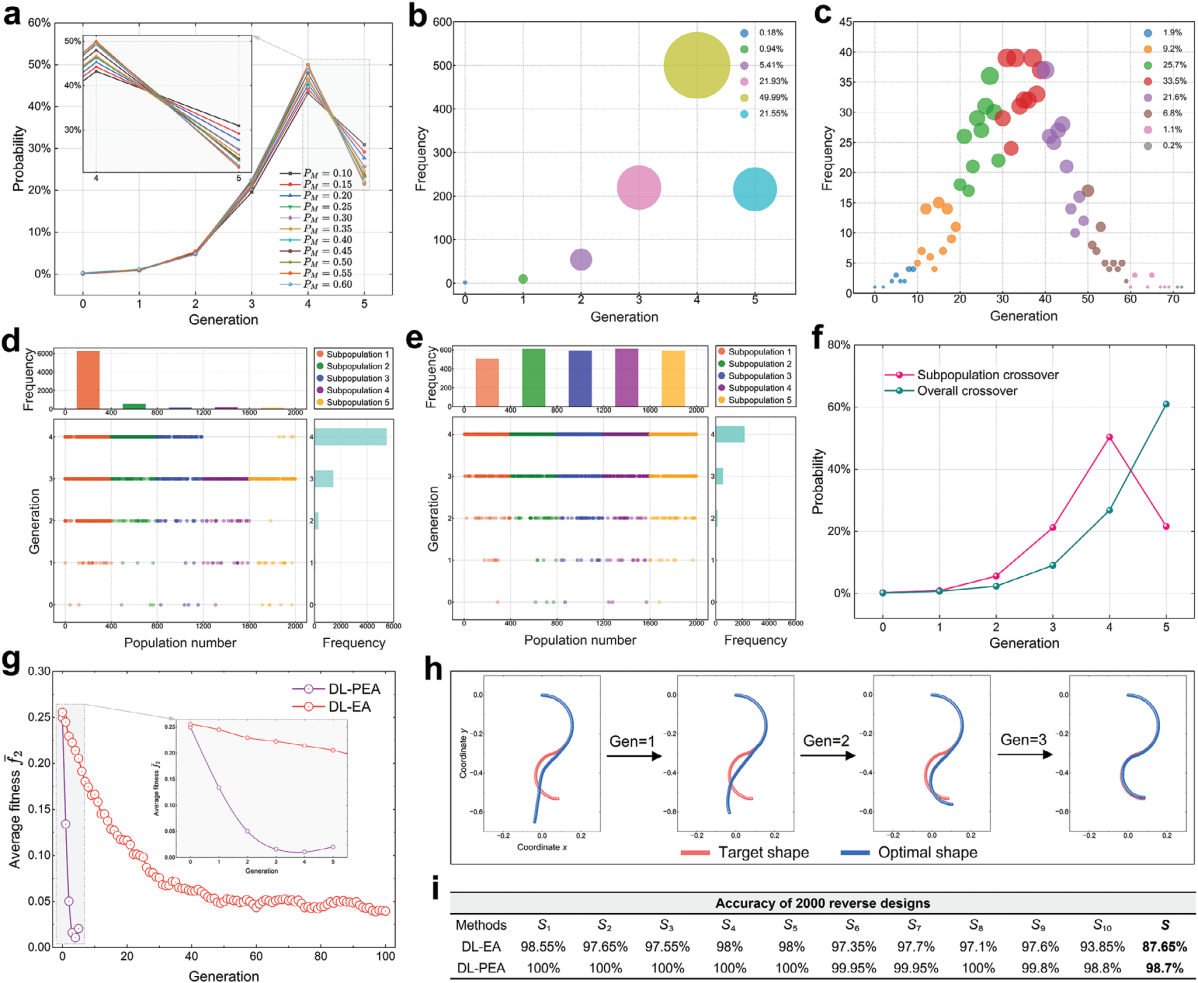
In‐depth analysis of DL‐PEA performance and performance comparison with DL‐based traditional EA (DL‐EA) method. a) Effect of different mutation probabilities on the DL‐PEA performance, with the best performance when the mutation probability is 0.55. b) Using DL‐PEA for 1000 reverse designs, the optimal design is observed in the fourth generation of evolution accounted for the highest proportion, reaching 50%. c) Using DL‐EA for 1000 reverse designs, the highest proportion of determining the optimal design by evolving 20–40 generations reached 80%. d) DL‐PEA performs 5000 reverse designs using subpopulation crossover, where the optimal design occurs with the highest frequency in subpopulation 1. e) DL‐PEA executes 5000 reverse designs employing an ungrouped overall crossover strategy, wherein the frequencies of the optimal design appearing across the five subpopulations are approximately equal. f) Performance comparison of the subpopulation crossover approach and the overall crossover approach demonstrated that the subpopulation crossover approach identifies the optimal design in fewer evolutionary cycles. g) Comparison between the changes in population average fitness over a series of evolutions for both DL‐PEA and DL‐EA. The results reveal that the population average fitness of DL‐PEA consistently surpasses that of DL‐EA (noting that a smaller ${\bar{f}}_{2}$ value indicates higher fitness). h) This example illustrates the rapid reverse design capability of DL‐PEA, which achieves the optimal design within only three evolutions. i) Using DL‐PEA and DL‐EA for 2000 reverse designs and comparing their design accuracies, the accuracy of DL‐PEA was significantly higher than that of DL‐EA.

We designate the final configuration of a voxelized hydrogel from FE simulation as the target shape and employ DL‐PEA for reverse design. This approach facilitates precise verification of whether the optimal design solution corresponds to the original voxel encoding, enabling a comprehensive performance analysis of the system. First, we investigated the impact of mutation probability on the efficacy of DL‐PEA in achieving rapid reverse design. Mutation probability, denoted as *P*_*M*_, is the likelihood that a voxel unit within the unidentified domain of each individual undergoes mutation, where mutation involves altering the voxel coding from 0 to 1 or vice versa. We conducted ten sets of experiments, each consisting of 1000 iterations, for various mutation probabilities. The average results from these ten sets were considered as the final outcome, as depicted in **Figure** **6**a. The findings indicate that the lowest proportion of DL‐PEA identifying the optimal design by the 5th evolution occurred at a mutation probability of 0.55. Therefore, a mutation probability of 0.55 enables the system to identify the optimal design with fewer evolutionary cycles, thereby enhancing efficiency. The result in Figure 6b illustrates that the DL‐PEA method can identify the optimal design solution for the target shape within a maximum of five evolutions, achieving a 50% probability of success by the fourth evolution. In contrast, the traditional DL‐EA method identifies the optimal design with DL‐EA peaks between 20 and 50 evolutions, reaching 80% (Figure 6c). This suggests that DL‐PEA is significantly more efficient, capable of finding the optimal design in a shorter timeframe.

To assess the effectiveness of grouping populations and then performing crossover within subpopulations, we conducted 5000 simulation experiments. The results are displayed in Figure 6d, where differently colored balls represent various subpopulations. Frequency statistics revealed that the occurrence of the optimal design in subpopulation 1 was significantly higher compared to other subpopulations. In contrast, Figure 6e illustrates the use of an overall crossover strategy without grouping, where offspring are numbered and categorized into five subpopulations. The data indicate that the frequency of the optimal design across these subpopulations was approximately equal. However, the subpopulation crossover method demonstrated a significantly higher frequency of achieving the optimal design during the first four evolutionary cycles compared to the overall crossover method. This comparative result suggests that the crossover between superior individuals is more likely to yield superior outcomes. Consequently, the subpopulation crossover approach facilitated the discovery of the optimal design in fewer evolutionary cycles than the overall crossover approach, as detailed in Figure 6f. Specifically, the subpopulation crossover method achieved the optimal design in 80% of cases within the first four evolutions, whereas the overall crossover method only reached a 40% success rate in the same time frame.

Figure 6g illustrates the variation in population mean fitness, ${\bar{f}}_{2}$, with the number of evolutions for the DL‐PEA and DL‐EA methods. Both methods were applied to 2000 reverse designs, and the results reveal that DL‐PEA consistently outperformed DL‐EA in terms of average fitness across all evolutions, indicating a more rapid improvement in fitness. Remarkably, DL‐PEA achieved an exceptionally high level of population‐averaged fitness within just four evolutions, whereas DL‐EA required ≈100 evolutions to attain a comparable fitness level. It is important to note that during the 5th evolution, DL‐PEA implemented a global traversal strategy to identify the optimal solution, which led to a slight reduction in the average fitness of the population. Figure 6h presents an example of reverse design using DL‐PEA. For a given target shape, DL‐PEA efficiently determines the optimal shape in each evolution by optimizing each column sequentially. By the 3rd generation, there is a high degree of congruence between the optimal shape and the target shape, demonstrating the exceptional efficiency of the method.

Figure 6i presents a comparison of the design accuracy between the DL‐PEA and DL‐EA methods across 2,000 reverse design experiments. In this context, *S*_*i*_ represents the i‐th column of the voxel unit within the hydrogel, whereas ***S*** refers to the overall structure of the hydrogel. The findings indicate that DL‐PEA consistently outperforms DL‐EA in terms of design accuracy, both at the level of individual voxel columns and across the entire structure. Specifically, DL‐PEA achieved an overall design accuracy of 98%, compared to 87.65% for DL‐EA. Notably, DL‐PEA attained 100% accuracy in designing the first five columns of voxel units, underscoring the effectiveness and precision of its column‐by‐column optimization approach.

Furthermore, we evaluated the time efficiency of the FE‐based PEA, the DL‐EA, and the proposed DL‐PEA by comparing their performance on a single reverse design. The results are presented in **Table** **2**. Considering the extensive duration required for FE simulations, the FE‐PEA method was tested on only one reverse design, which required 180 h. In contrast, the average time for a single reverse design using the DL‐EA method was 20.8 s, whereas the DL‐PEA method achieved a significantly lower average of 3.04 s. This comparison underscores the substantial efficiency advantage of the proposed DL‐PEA method in reverse design processes, offering a highly efficient solution for the reverse optimization of voxelized composite structures.

**Table 2:** Time consumption for a single reverse design using three methods (FE‐PEA, DL‐EA, and DL‐PEA), “h” for hours and “s” for seconds. FE‐PEA method performed one reverse design, DL‐EA and DL‐PEA performed 2000 reverse designs, and the respective averages were obtained as the final results.

Reverse design methods	Time consumption
FE‐PEA	180 h
DL‐EA	20.8 s
DL‐PEA	3.04 s

### 2.6 Fast Reverse Design of Biological Skeletal Lines and Hand‐drawn Curves

**Figure 7:**
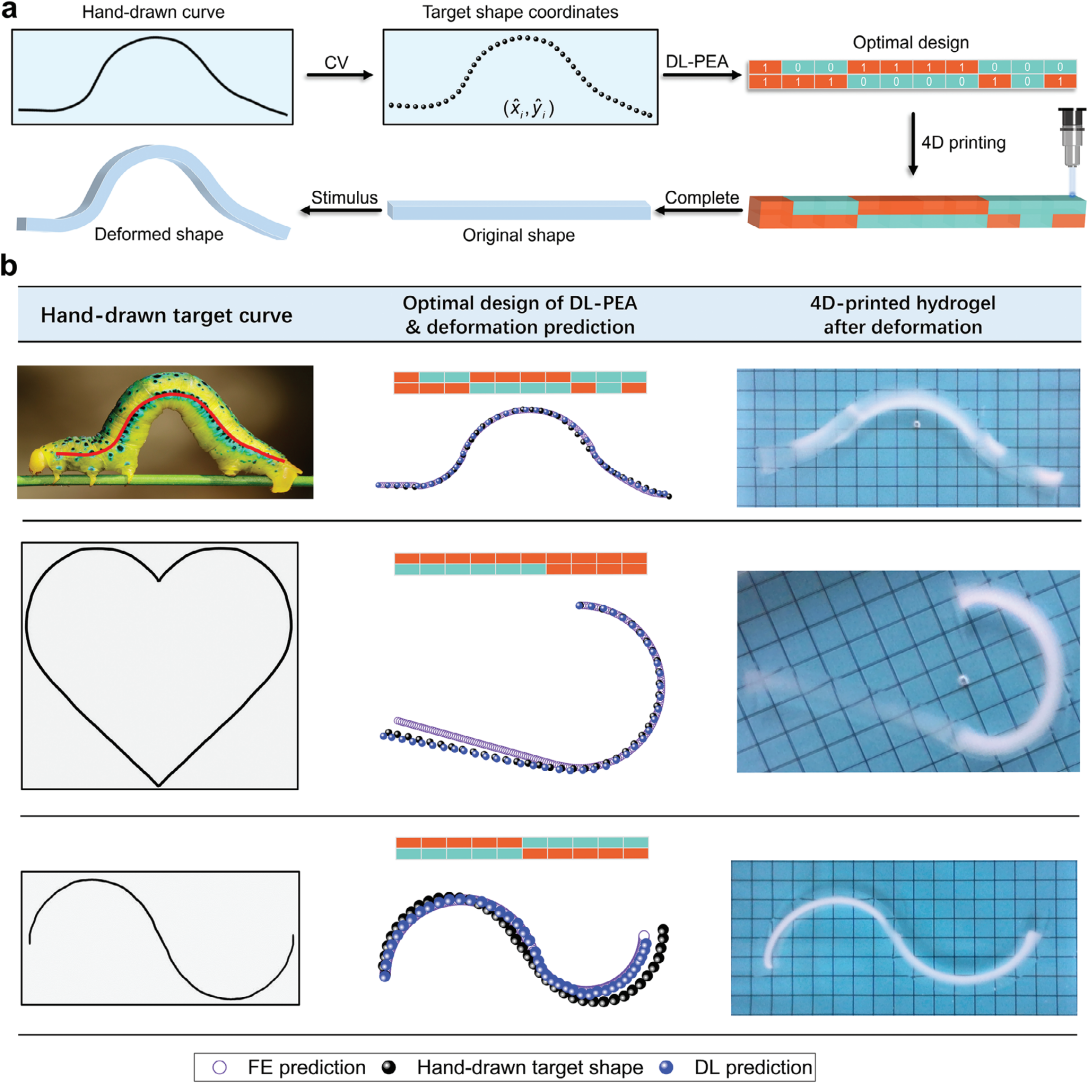
DL‐PEA enables rapid reverse design of hand‐drawn curves, integrated with 4D printing to facilitate a comprehensive design and manufacturing process. a) The complete 4D printing design and manufacturing sequence involves: CV recognizes target curves to generate coordinates, DL‐PEA determining the optimal design, 4D printing of hydrogel based on this design, and environmental stimuli inducing hydrogel deformation. b) An example of the reverse design process for hand‐drawn curves is illustrated as follows: The first column displays three shapes–a wriggling caterpillar, a heart, and a sinusoidal curve. The second column presents the optimal design solutions and deformation prediction results achieved by DL‐PEA. The third column depicts the actual deformation outcomes of the hydrogel after it absorbs and swells in water.

The high accuracy and efficiency of the proposed DL‐PEA reverse design method have been verified by simulations. This method is combined with 4D printing technology to facilitate the entire design and manufacturing process, as depicted in **Figure** **7**a. First, computer vision algorithm is employed to accurately identify the contours of hand‐drawn curves and to generate the coordinates of the desired shape. Subsequently, the DL‐PEA method determines the optimal design solution for the desired shape, specifically the optimal distribution of material properties. Finally, the final design is constructed layer‐by‐layer using 4D printing technology. In this process, units encoded with 1 are UV‐scanned three times, whereas the units encoded with 0 receive only one scan. The resulting hydrogel formes a beam‐shaped structure. Upon absorbing water and expanding, the deformation of the structure is strongly consistent with the designed target shape. Details about image recognition and coordinate generation for hand‐drawn curves are shown in Figure S6, (Supporting Information).

This study focuses on the deformations of voxelized composite structures. To demonstrate the reliability of the proposed DL‐PEA method in inverse design, we utilized 4D printing technology to produce the optimal design models of the target shapes. The printed hydrogel sample was placed in purified water, allowing it to swell by absorbing water until reaching the maximum deformation. Then, the deformed shape of the hydrogel was compared with the target shape and the FE simulation results to evaluate consistency, thereby verifying the effectiveness of the design method. Figure 7b demonstrates reverse designs for three distinct shapes. The first column exhibits the hand‐drawn target curves, the second column reveals the optimal inverse design and deformation prediction for each shape, and the third column displays the deformation results for the 4D‐printed voxelized hydrogel. The initial design objective was to replicate the shape of a wriggling caterpillar. The second design objective focused on a heart shape. Owing to the angular constraints of material deformation and limitations in print size, the specific target was a half‐heart shape. The third design aimed to achieve a sinusoidal curve, and the DL‐PEA method successfully identified the optimal configuration. The simulation results confirmed that the predicted deformation aligns with the target shape. Following the reverse design process, the material properties were specified, and the object was printed using the optimal design. The printing results show that the deformed shape of the hydrogel is highly consistent with the target shape. Furthermore, the actual deformation of the hydrogel is consistent with the FE simulation results, demonstrating that the material property distribution, precisely designed through 4D printing, can achieve stable stimulus responses in real‐world environments. In the hydrogel, the transparent sections correspond to the units encoding “1,” whereas the white sections represent the units encoding “0.” This color differentiation results from varying polymerization degrees within the hydrogel. All three shapes of the hydrogel were successfully printed in a single attempt, and the deformed shapes were generally consistent with the target shapes, demonstrating a high success rate and indicating the reproducibility and stability of the experiment. These instances further highlight the effectiveness and adaptability of the DL‐PEA method as a rapid reverse design tool capable of accurately configuring material properties for complex shapes and having high reliability in deformation control.

## 3 Discussion

In this study, we successfully implement a fast reverse design of 4D printed voxelized composite structures by integrating DL and EA optimization techniques. We utilize an intelligent response hydrogel as the optimization target to create voxelized structures through 4D printing technology. The SEP‐CNN deep learning model and its corresponding loss function were developed to accurately and efficiently predict the deformation of the hydrogel. The rapid prediction capabilities of the SEP‐CNN model are crucial for achieving swift reverse design, which is unattainable with the traditional FE method. The traditional FE method requires several minutes to complete a single simulation, whereas a single reverse design process requires thousands of simulations, leading to significant time expenditure. In terms of structural characteristics, the proposed PEA method employs a column‐by‐column optimization strategy to determine the optimal material distribution for the target shape with fewer evolutions, thus enhancing the efficiency of the reverse design process. Consequently, the DL‐PEA framework, which combines DL and PEA, demonstrated remarkable efficiency in the reverse design of target shapes, completing the reverse design of a single target shape in only 3.04 s.

The reverse design accuracy of the DL‐PEA method reached 98.7%, significantly surpassing the 87.65% accuracy of the traditional DL‐based EA method. Consequently, the DL‐PEA method enhances reverse design efficiency and accuracy, which are crucial for implementing batch reverse design. Furthermore, the DL‐PEA method has wide applicability, extending beyond specific hydrogel materials to other non‐mechanical systems such as liquid crystal and liquid crystal elastomer materials. This method can optimize the molecular alignment and liquid crystal phase of liquid crystal materials, thereby achieving precise performance control. It can also accommodate more complex structures or multi‐property material systems through model expansion. Thus, this method offers an effective tool for optimizing 4D‐printed voxelized composite structures, facilitating the practical application of 4D printing. This method allows for quicker and more cost‐effective design iterations in 4D printing, potentially accelerating the production and innovation cycles in industries like biomedical engineering, smart textiles, and soft robotics.

## 4 Experimental Section

### Preparation of Hydrogel Materials

The DMAAm used for synthesizing hydrogels was sourced from Tokyo Kasei Kogyo Co. (Tokyo, Japan). The polymer HPC was obtained from Wako Pure Chemical Industries, Ltd. (Osaka, Japan). The crosslinker KarenzMOI‐EG was procured from Showa Denko K.K. (Tokyo, Japan). Both hydrophobic and hydrophilic initiators, TPO, were acquired from Tokyo Kasei Company, Japan. The UV absorber AS150 was purchased from Nippon Kayaku Co. (Tokyo, Japan). The 4D printer was supplied by Yorokobi Ltd. (Yamagata, Japan).

To ensure structural stability during printing and response, the formulation (Table S1, Supporting Information) and configuration process were strictly followed to prepare the hydrogel solution. To fabricate the hydrogels, HPC powder was initially dissolved in a DMAAm solution and stirred in a blender for one day to ensure complete dissolution. Following this, the crosslinker KarenzMOI‐EG was added, and the mixture was stirred for 30 min to facilitate the chemical reaction. This crosslinker not only significantly enhances the internal crosslinking strength of the hydrogel through chemical reaction but also exhibits good adhesiveness, which promotes bonding between material surfaces. During the printing process, the crosslinker can enhance the adhesion between hydrogels and ensure the stability of the structure. Then, the reaction was halted by adding purified water to the mixture. Subsequently, the initiator TPO and the UV absorber AS150 were incorporated, and the mixture was stirred again for 30 min to prepare the hydrogel solution. Under UV light, the solution underwent a light‐initiated polymerization reaction, forming a solid hydrogel with a cross‐linked structure. The intensity, rate, and number of UV irradiation cycles were adjusted to control the degree of hydrogel polymerization. In the 4D printing process of hydrogels, by controlling the degree of polymerization within each voxel region, structures with different cross‐linking densities can be constructed, resulting in hydrogel structures with swelling gradients.

The 4D printing equipment used in this study was the first generation of printers developed by Yamagata University. The introduction of the printer can be seen in Figure S9 (Supporting Information). During the printing process, structural design was achieved by precisely controlling the swelling rate of each voxel unit. Specifically, voxel units encoded as “1” underwent three UV scans, resulting in a higher swelling rate, while those encoded as “0” were scanned only once to maintain a lower swelling rate. **Table** **3** shows the parameter values of UV light. After printing, the hydrogel samples were placed in purified water for swelling tests under standard atmospheric pressure and room temperature of 25 °C. The deformation behavior was observed to determine whether it was consistent with the FE simulation results.

**Table 3:** Parameter values of UV light used for 4D printing.

Parameter of UV light	Value
Wavelength	405 nm
Scanning speed	50 mm s^−1^
Spot diameter	0.3 mm
Intensity	10 mW

### Generation of Datasets by FE Simulation

Simulations were conducted using the professional FE analysis software ABAQUS (version 2023) to generate datasets necessary for the DL model. The entire modeling and simulation process was executed by running Python scripts within ABAQUS. The hydrogel model was designed in the shape of a beam, with dimensions of 100 mm in length, 5 mm in width, and 5 mm in thickness. It was segmented into a grid of two rows and ten columns (2 × 10), with each voxel unit measuring 10 mm × 5 mm × 2.5 mm. Consequently, this hydrogel structure can be configured in 2^20^ (≈1.05 × 10^6^) different possibilities. Utilizing the NumPy library, 10,000 sets of (2 × 10) binary encodings were created and assigned to the corresponding voxel units for FE simulation. The simulation modeled the water‐absorbing expansion behavior of the hydrogel through thermal expansion. The coefficient of thermal expansion for the active material, encoded as “1,” was set at 0.021223, whereas for the passive material, encoded as “0,” it was set at 0.018938. To simulate the mechanical response to water absorption and expansion, the temperature of the model was incremented from 0 to 100 °C, with a maximum temperature change of 15 °C per increment. At 100 °C, the active material demonstrated a linear expansion of 2.1223 times its original size, and the passive material expanded by 1.8938 times.

Following the simulation, 200 nodes along the deformation trajectory from the center of the hydrogel were selected as deformation coordinate points. These points, along with the material property encodings, served as the input and output data for the DL model, respectively. Optimal training results were achieved with a sample size of 8000, which was divided into training and testing sets in a 0.75 to 0.25 ratio. A detailed description of the dataset is available in Figure S1 (Supporting Information).

### DL Model Parameters and Loss Function Design

The SEP‐CNN model designed for this study was compared with traditional CNN and LSTM models, all constructed using the TensorFlow (version 2.5) framework. During the training process, the model was optimized using the Adam optimizer with a fixed learning rate of 10^−4^ and a batch size of 60. The inputs were binary encoded without normalization, whereas the outputs underwent normalization via scale scaling. Initially, the training involved 100 steps per epoch, where each step consisted of processing a batch of data. For each subsequent epoch, the number of training steps increased by 100, and the training was completed in 80 epochs.

For the prediction task in this work, the mean square error (MSE) is commonly employed as the loss function for training models. MSE measures the discrepancy between the predicted values and the actual values.

(3) $$\begin{array}{c} L_{MSE}=\frac{1}{N}\sum_{i=1}^{N}\left[{\left(x_{i}-{\hat{x}}_{i}\right)}^{2}+{\left(y_{i}-{\hat{y}}_{i}\right)}^{2}\right] \end{array}$$

Although MSE is a commonly employed method to measure the difference between predicted and true values, using only *L*_*MSE*_ as a loss function may not capture all the details in hydrogel deformation prediction, thus affecting prediction accuracy. To enhance prediction accuracy, two new metrics were introduced: the adjacent distance squared error (ADSE) and the angle cosine squared error (ACSE). These metrics aim to refine the alignment of predicted points with respect to the true trajectory by addressing the limitations of *L*_*MSE*_.

Denote the distance between two adjacent true points as $I=\{I_{1},I_{2},\text{…},I_{N-1}\}$ and the distance between two adjacent predicted points as $\hat{I}=\left\{{\hat{I}}_{1},{\hat{I}}_{2},\text{…},I_{N-1}\right\}$, and ADSE is defined as follows:

(4) $$\begin{array}{c} ADSE=\sum_{i=1}^{N-1}{\left(I_{i}-{\hat{I}}_{i}\right)}^{2} \end{array}$$

Denote the angle cosine formed by three adjacent true points as cos θ = {cos θ_1_, cos θ_2_, ⋅⋅⋅, cos θ_*N* − 2_} and the angle cosine of three adjacent predicted points as $\cos\hat{\theta }=\left\{\cos{\hat{\theta }}_{1},\cos{\hat{\theta }}_{2},\text{…},\cos{\hat{\theta }}_{N-2}\right\}$, and ACSE is defined as follows:

(5) $$\begin{array}{c} ACSE=\sum_{i=1}^{N-2}{\left(\cos\theta_{i}\cdot \frac{I_{i}\cdot I_{i+1}}{I_{i}+I_{i+1}}-\cos{\hat{\theta }}_{i}\cdot \frac{{\hat{I}}_{i}\cdot {\hat{I}}_{i+1}}{{\hat{I}}_{i}+{\hat{I}}_{i+1}}\right)}^{2} \end{array}$$

The total loss function of the model can be constructed by combining the main loss function MSE with the auxiliary loss functions ADSE and ACSE and assigning varying weights to them:

(6) $$\begin{array}{c} L_{total}=L_{MSE}+\alpha L_{ADSE}+\beta L_{ACSE} \end{array}$$

where α, β denote the weighting factors. Note that the ACSE does not directly evaluate the variance of angle‐cosine values as a metric. This is because the squared values of the angle cosines were significantly larger than the values of MSE and ADSE during training. Simply scaling and incorporating it into the total loss function did not effectively enhance the model performance. More importantly, a stepwise strategy was adopted during the optimization process. Initially, MSE was optimized, and once its value falls below a specified threshold, ADSE and ACSE were progressively included in the optimization objectives. To this end, a dynamic scaling method, i.e., $\cos\theta_{i}\cdot \frac{I_{i}\cdot I_{i+1}}{I_{i}+I_{i+1}}$ and $\cos{\hat{\theta }}_{i}\cdot \frac{{\hat{I}}_{i}\cdot {\hat{I}}_{i+1}}{{\hat{I}}_{i}+{\hat{I}}_{i+1}}$ was used in Equation (5). As the training data were normalized to 0 < *I*_*i*_, *I*_*i* + 1_ < 1, $0<\frac{I_{i}\cdot I_{i+1}}{I_{i}+I_{i+1}}<I_{i},I_{i+1}<1$ could be taken into consideration. The simulations verified that this scaling approach ensures that the values of ACSE, ADSE, and MSE were approximately on the same order of magnitude. Moreover, this approach aligns with the stepwise optimization strategy. Specifically, when the value of ADSE was optimized to a negligibly small value, it could be approximated that $\frac{I_{i}\cdot I_{i+1}}{I_{i}+I_{i+1}}\approx \frac{{\hat{I}}_{i}\cdot {\hat{I}}_{i+1}}{{\hat{I}}_{i}+{\hat{I}}_{i+1}}$. At this point, the optimization focus in ACSE primarily shifts to the angle cosine value. Therefore, when ADSE is optimally minimized, ACSE became effectively significant. The values of the weighting factors α and β are crucial in this stepwise optimization strategy. A dynamic weight adjustment method was designed, and the values of α and β are determined by Equation (7).

(7) $$\left(\alpha ,\beta \right)=\left\{\begin{array}{cc} \left(0,0\right), & \;epoch⩽7\\ \left(\frac{{\bar{MSE}}_{\text{epoch}-1}}{15{\bar{ADSE}}_{\text{epoch}-1}},\frac{{\bar{MSE}}_{\text{epoch}-1}}{150{\bar{ACSE}}_{\text{epoch}-1}}\right), & \;epoch>7 \end{array}\right.$$

where ${\bar{MSE}}_{epoch-1}$, ${\bar{ADSE}}_{epoch-1}$, and ${\bar{ACSE}}_{epoch-1}$ represent the average values of MSE, ADSE, and ACSE in the preceding epoch, respectively. In the optimization process, the primary objective was to minimize the discrepancy between predicted and true values; therefore, MSE is prioritized. Consequently, the weights α and β were initially set to zero, i.e., for *epoch* ⩽ 7. As the training progresses beyond *epoch* > 7 and the MSE value drops below 10^−4^, ADSE and ACSE are gradually incorporated into the optimization objectives. In the total loss function, *L*_*MSE*_ remains the main optimization target, whereas *L*_*ADSE*_ and *L*_*ACSE*_ serve as auxiliary objectives, guiding the predicted values to align with the trajectory of the true values. Thus, they were auxiliary optimization goals. More detailed theory on the design of the loss function is illustrated in Figure S3 (Supporting Information).

### Design of the DL‐PEA Framework

In this study, a PEA tailored for the sequential characteristics of voxelized hydrogels was developed. This algorithm efficiently identifies the optimal design using a column‐by‐column optimization strategy, as detailed in Figure 5. Each iteration of the population evolution employs a fitness function, denoted as *f*_1_, to select high‐performing individuals from a superior population. The selection mechanism evaluates the fitness of a specific column during each evolutionary step. In each column, there were four potential encoding forms. The encoding that yields the highest average fitness was selected by computing the average fitness across these four encodings. Individuals with this optimal column encoding are then included in the excellent population. When the population evolves to the fifth generation, the optimal local encoding of the first five columns is entirely determined. This column‐by‐column optimization strategy effectively avoids falling into local optimal solutions during the optimization process, significantly improving the efficiency and accuracy of inverse design compared to traditional EA.

In each evolutionary step, 100 elite individuals with the highest fitness were also retained, and these elite individuals participate in crossover and mutation operations, further preventing local optimal solutions. Even if the population did not find the optimal solution by the fifth generation, the first five columns were already fixed. With 1024 possibilities remaining for the undecided encodings, all possibilities could be evaluated to determine the optimal solution quickly. This approach effectively avoids falling into local optimal solutions. However, this requires that the prediction accuracy of the DL model be extremely high, which was an essential indicator in the design of the DL model. For structures with more voxel units, the specific evolution strategy of the PEA was determined on a case‐by‐case basis.

## Conflict of Interest

The authors declare no conflict of interest.

## Author Contributions

M.W., Z.W., R.X., and L.M. conceived the idea and designed the study. M.W., Z.L., H.F., and Z.Q. synthesized the hydrogel. M.W., Z.L., and Y.T. performed the modeling and algorithm design. M.W., Z.L., Y.G., and Y.X. performed the 4D printing and hydrogel preparation. M.W. collected the dataset and wrote the manuscript. All authors discussed the results and evaluated the manuscript.

## Supporting information

Supporting Information

## Data Availability

The data and code are available at http://www.ihpc.se.ritsumei.ac.jp/obidataset.html or https://github.com/MengtaoWANG/Fast‐reverse‐design‐of‐4D‐printing.
