# Supplementary material for: Fast Reverse Design of 4D‐Printed Voxelized Composite Structures Using Deep Learning and Evolutionary Algorithm
Source: Adv Sci (Weinh). 2025 Feb 1;12(12):2407825. doi: 10.1002/advs.202407825 (PMC11948029; doi:10.1002/advs.202407825)
Supplement: Supplementary file 1 — Supporting Information [file ADVS-12-2407825-s001.pdf]

ADVANCED SCIENCE
Open Access

# Supporting Information

for *Adv. Sci.*, DOI 10.1002/advs.202407825

Fast Reverse Design of 4D-Printed Voxelized Composite Structures Using Deep Learning and Evolutionary Algorithm

*Mengtao Wang, Zaiyang Liu, Hidemitsu Furukawa, Zhuo Li, Yifei Ge, Yifan Xu, Zhe Qiu, Yang Tian, Zhongkui Wang*, Ren Xu* and Lin Meng**

# Supporting Information

## Fast reverse design of 4D-printed voxelized composite structures using deep learning and evolutionary algorithm

Mengtao Wang, Zaiyang Liu, Hidemitsu Furukawa, Zhuo Li, Yifei Ge, Yifan Xu, Zhe Qiu, Yang Tian, Zhongkui Wang,* Ren Xu,* and Lin Meng*

**This PDF file includes:**
Figure S1 to S10, Table S1.

Table S1 shows the material formulation of hydrogel. To ensure structural stability during printing and response, the hydrogel solution is prepared strictly following the formulation and preparation process. The material dosage is accurately controlled to 5 decimal places to ensure that the material properties of each batch solution are consistent.

**Table S1.** Hydrogel material formulation.

| Number | Material Name | Dosage(g) |
|---|---|---|
| 1 | DMAAm | 396.52 |
| 2 | HPC | 3.6032 |
| 3 | KarenzMOI-EG | 3.984 |
| 4 | TPO | 5.2257 |
| 5 | Purified water | 578.40 |
| 6 | Absorber | 1.97547 |

The composition of the dataset has a certain impact on the performance of the DL-based prediction model. To this end, we perform a statistical analysis of the training dataset and the testing dataset. Figure S1 (a, d) show all the deformation curves in the training and testing datasets, and these curves extend outward from the coordinate origin. Figure S1 (b, e) show the scatter density plots of the deformation curves corresponding to the first two columns of voxel units in the training and testing datasets. Because each column has four different encodings, the curves corresponding to the voxel units in the first column have four different bending directions, and the second column has sixteen bending directions. As the number of columns increases, the possibilities of bending directions expand four times. Therefore, excessive arbitrariness should be avoided when drawing the initial segments of the curve. Figure S1 (c, f) show the scatter density plots of the deformation curves of the last column of voxel units in the training and testing datasets, showing the consistency between the two datasets and indicating that they both follow random distribution rules.

In addition, the number of 0 and 1 encodings in the datasets are also counted: the proportions of 0 and 1 in the training dataset are 49.945% and 50.055%, respectively, and the proportions of 0 and 1 in the testing dataset are 50.23% and 49.77%, respectively. Among a total of 8,000 samples, only 30 samples are duplicates. The statistical results indicate that both the training and testing datasets follow a random distribution pattern, with a balanced representation of encodings. This ensures that the model can better learn the general characteristics of the data, rather than overfitting to specific patterns, thereby enhancing its generalization ability.

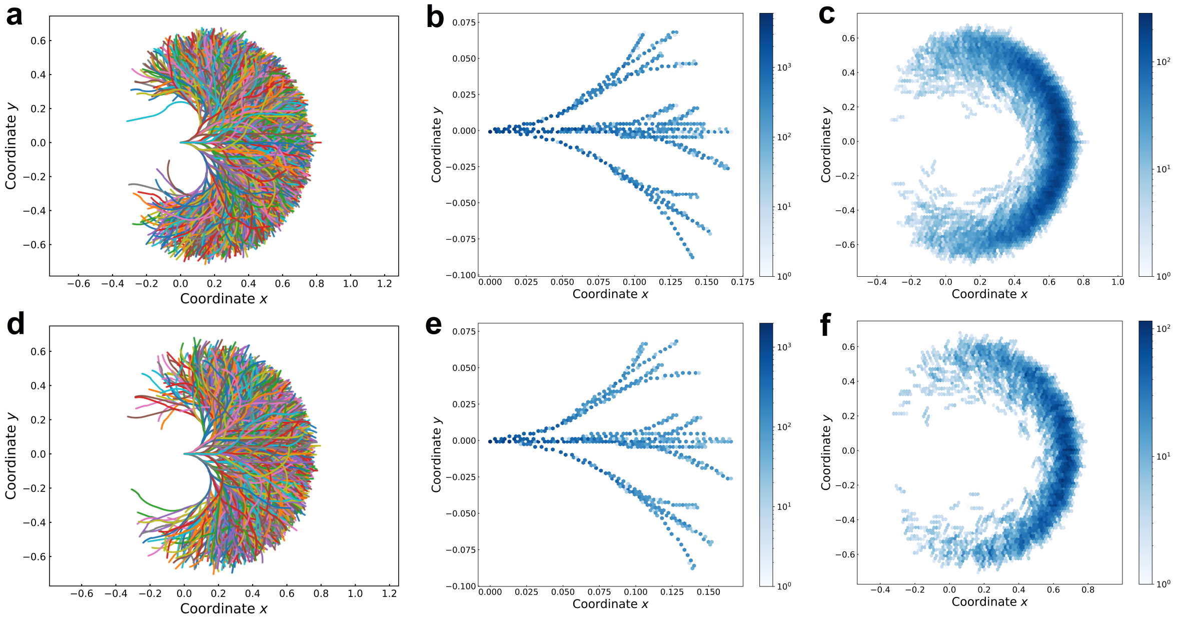


**Figure S1**. Statistical analysis of training and test datasets. (a, d) All the deformation curves of the training and testing datasets. (b, e) The deformation coordinates of the first two columns of voxel units in the training and testing datasets. (c, f) The deformation coordinates of the last column voxel units in the training and testing datasets.

Although MSE is a commonly employed method to measure the difference between predicted and true values, using only $L_{MSE}$ as a loss function may not capture all the details in hydrogel deformation prediction, thus affecting prediction accuracy. Considering Figure S3 b as an example, where Figure S3 (I) represents the ideal prediction result, and Figure S3 (II, III, and IV) exhibit three unideal prediction results that neglected details may cause. Given that each true value in our dataset is accurate to six decimal places, it is challenging for the predicted values to match the true values exactly. We propose using the average extreme distance (AED), which quantifies the mean distance between all predicted coordinate points and the actual coordinate point, denoted as $I_{AED}$. This measure could potentially provide a more comprehensive assessment of prediction accuracy. In Figure S3 (I), the distance between the predicted and true values equals $I_{AED}$, and the distances between two adjacent predicted points match those between two adjacent true points, i.e., $I_1 = \hat{I}_1$ and $I_2 = \hat{I}_2$. Additionally, the angle formed by any three consecutive predicted points is identical to that formed by the corresponding true points, denoted as $\theta_1 = \hat{\theta}_1$. This implies that the predicted and actual coordinate points align along the same trajectory, analogous to a railroad track. Thus, Figure S3 (I) represents an ideal prediction result. In contrast, Figure S3 (II, III, and IV) also portray that the distance between the predicted and true values reaches $I_{AED}$. However, discrepancies may occur in the distances between adjacent points or in the angles formed by three consecutive points in the predicted and true values. This variation arises because the mean–squared error ($L_{MSE}$) primarily constrains the distances between predicted and true values, but does not control the distances or angles between adjacent points.

In evolutionary algorithms, the main purpose of crossover is to promote population diversity and to broaden the potential for exploring new solutions in the search space, thus enhancing the possibility of finding the global optimal solution. Variation increases the genetic diversity of the population by introducing small random changes and helps to avoid the algorithm from falling into local optima. Specific details about crossover and mutation in the proposed progressive evolutionary algorithm (PEA) are shown in Figure S4. Before performing the population crossover, the algorithm first sorts and groups the populations from high to low according to the fitness of the individuals. In this case, subpopulation 1 contains the best individuals, while the other subpopulations contain relatively suboptimal individuals. Given the variation among the different subpopulations, we adopt a differentiated crossover strategy. For the best subpopulation 1, as shown in Figure S4 a, the identified domain does not participate in the crossover, and the crossover occurs in the unidentified domains.

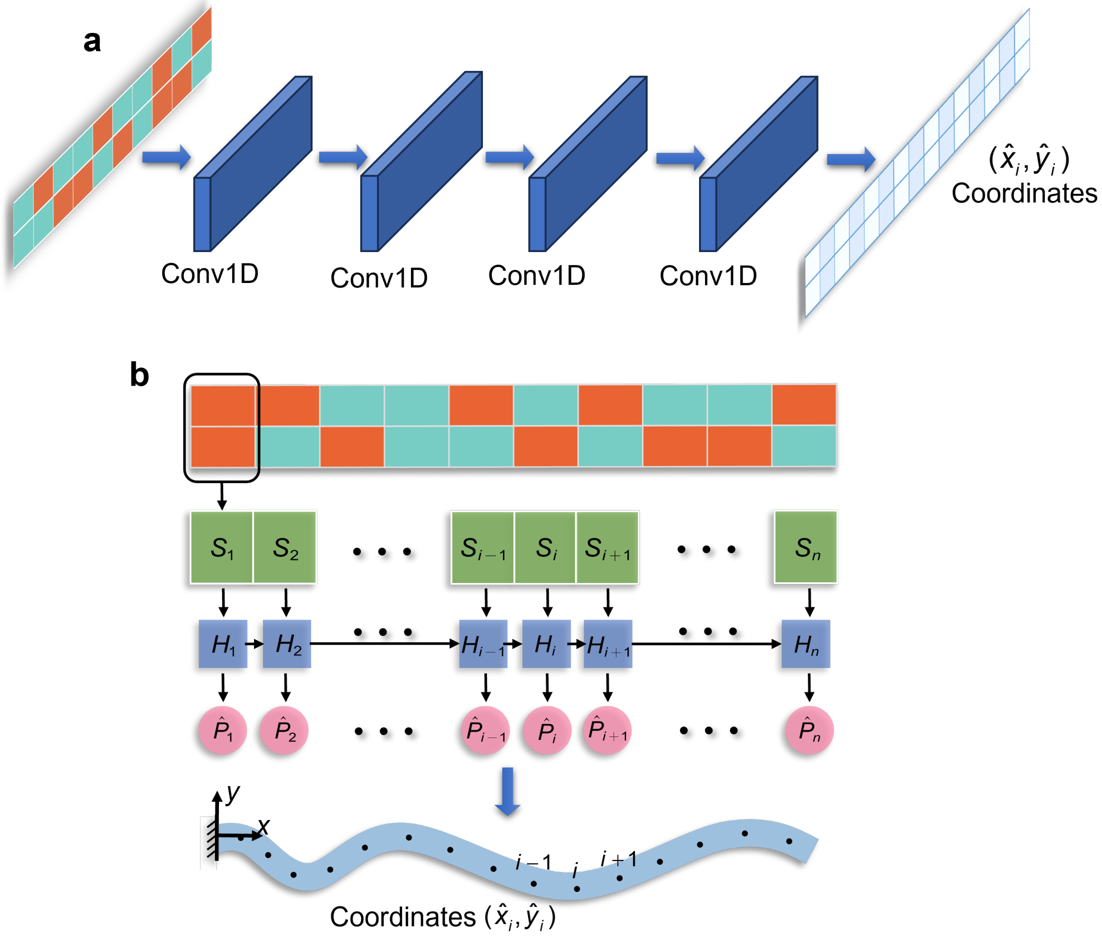


**Figure S2**. Schematic of CNN and RNN model architecture. (a) Traditional CNN model structure. The input of the model is a voxelized encoding of the hydrogel. The input is processed through four one-dimensional convolutional layers (Conv1D). Each convolutional layer operates to extract spatial features of the input data to construct an understanding of the voxelized structural response. Ultimately, the model outputs coordinate data of the deformed hydrogel. (b) RNN model structure consists of four LSTM layers. The voxel encoding is divided into $S_n$ cells, each representing a voxel data column. These units are inputted into the network one by one in chronological order. The output of each time step of the network $(H_i)$ depends not only on the input of the current time step, but also on the output of the previous time step $(H_{i-1})$, thus capturing the temporal dependence in the sequence data. Eventually, the model outputs the coordinate data of all time steps, which are combined to form the deformation curve of the hydrogel.

Considering that the first few columns of encodings in the undetermined domain of the best populations have a high probability of matching the target encodings, simply exchanging the last few columns of parental encodings is not effective in enhancing population diversity. For the suboptimal populations (subpopulations 2, 3, 4, 5), considering that the last two columns of individuals in the populations exhibit strong randomness, to enhance population diversity, the crossover method in Figure S4 b is adopted. In this method, neither the identified domain nor the first columns of the unidentified domain are involved in the crossover. This crossover method enhances the genetic diversity of the second and third columns of undetermined domains in the offspring, providing a broader space for exploration in the subsequent evolutionary process. With this differentiated crossover strategy, the progressive evolutionary algorithm can more effectively utilize genetic diversity, thereby improving the quality of the solution and the overall performance of the algorithm.

The mutation operation is divided into two stages, starting with a fixed-point mutation operation, as shown in Figure S4 c. The fixed-point mutation is performed for the first column of the undetermined domain, i.e., the column that needs to be optimized in the next evolution, to ensure that the

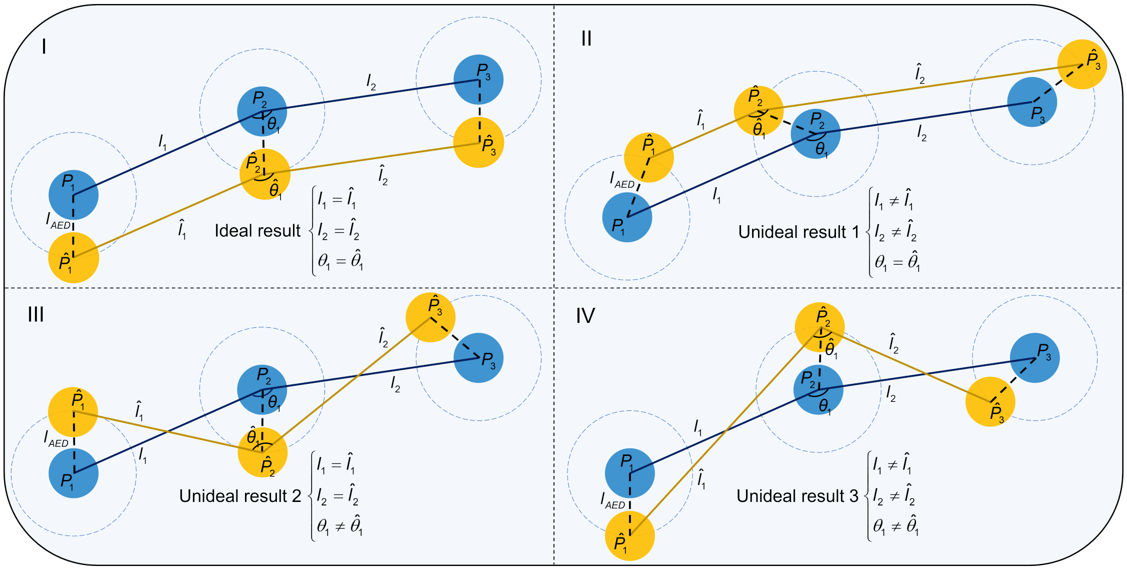


**Figure S3**. Loss function design concept. Taking three adjacent coordinate points as an example: The blue points are the real coordinate points, and the yellow points are the predicted coordinate points. Figure I shows the ideal prediction result. Figures II, III, and IV show three unideal results.

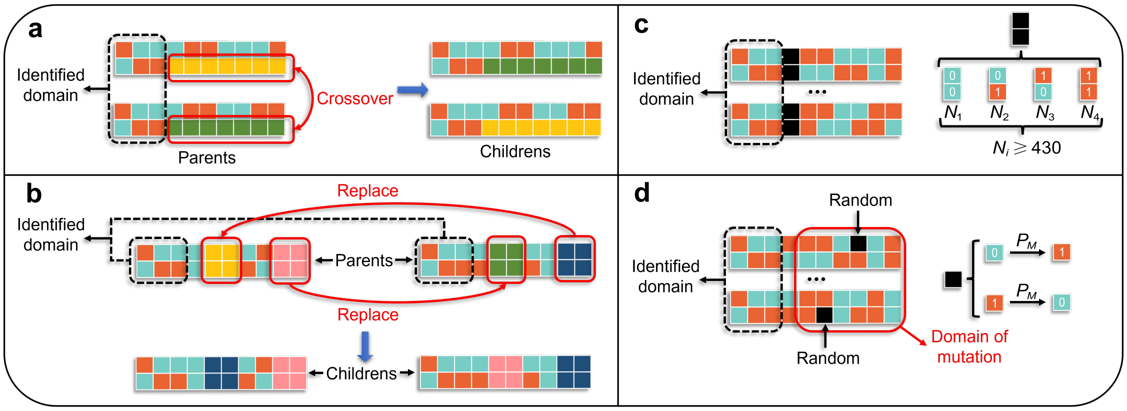


**Figure S4**. Specific details of crossover and mutation in the PEA reverse design method. (a) The crossover method of the best subpopulation 1. (b) The crossover method of other subpopulations. (c) Fixed point mutation operation. (d) Random mutation operation.

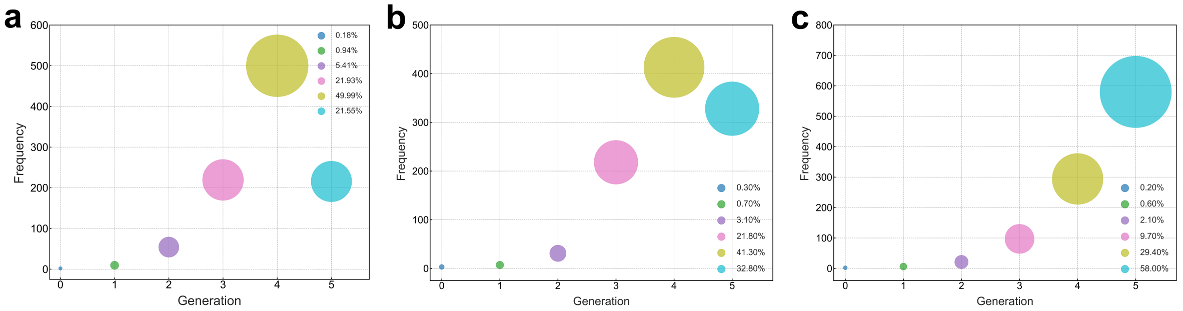


**Figure S5**. The comparison results of PEA using different crossover strategies for Reverse Design. (a) PEA uses the differentiated crossover strategy for 1000 reverse designs, and the probability of finding the optimal design at the fifth evolution is 21.55%. (b, c) PEA uses a single crossover approach for 1000 reverse designs, and the probability of finding the optimal design at the fifth evolution is 32.80% and 58%, respectively, with performance decreasing compared to the differentiated crossover strategy.

four encodings of this column have a balanced number of individuals, thereby achieving a balanced calculation of the average fitness. Fixed-point variation ensures that the number of per encoding is no less than 430.This not only ensures that the sample number is balanced when computing the average fitness for each encoding, but also ensures a sufficient number of individuals among subpopulations when grouping excellent populations. Setting the number of individuals to at least 430 means that in the next evolution of the excellent population group, the number of individuals in subpopulation 5 will be no less than 30. Among these 30 individuals, randomly selecting two individuals for crossover can produce 435 combinations, thus ensuring that the offspring population generated after crossover has sufficient diversity. After completing the fixed-point mutation, the population will undergo a random mutation operation, as shown in Figure S4 d. At this stage, except for the first column of the unidentified domain, any voxel unit in the unidentified domain will mutate with probability P, i.e., the encoding changes from 0 to 1 or from 1 to 0. This random mutation helps to introduce additional genetic diversity that enhances the exploration of the population and fuels the algorithm to find the optimal solution in a complex search space.

In the proposed PEA method, we employ a differentiated crossover strategy. To verify the superiority of this strategy, we conducted a comparison experiment, and the results are shown in Figure S5. Figure S5 a illustrates the results of using the differentiated crossover strategy, i.e., the approach depicted in Figure S4. Figure S5 (b, c) show the results for all subpopulations using only the single crossover method described in Figure S4 (a, b), respectively. Each crossover strategy undergoes 1000 reverse design experiments. The results show that the probability of finding the optimal design in the first four evolutions using the differentiated crossover strategy is as high as 78.45%. In contrast, the probability of obtaining the optimal design in the first four evolutions is only 67.2% and 42% for the approach using a single crossover strategy. This result indicates that adopting different crossover methods according to the characteristics of different subpopulations can significantly improve the efficiency of reverse design.

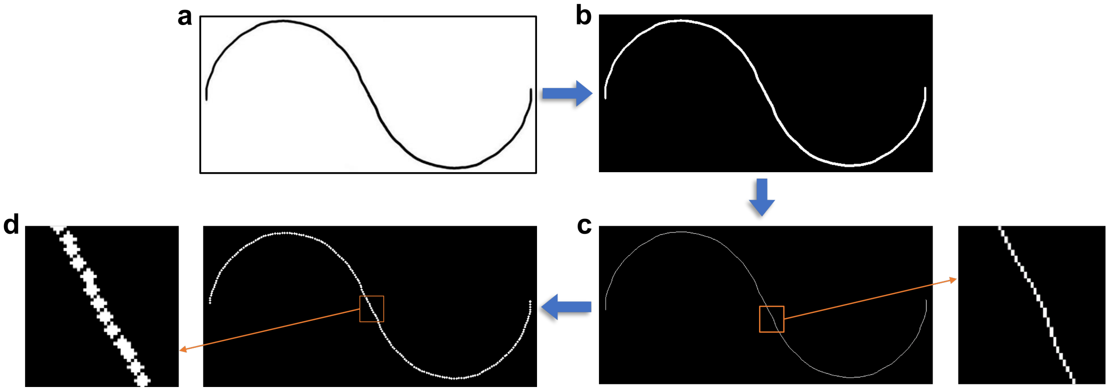


**Figure S6**. **Hand-drawn curve recognition and coordinate generation.** (a) Original image of the hand drawn curve. (b) Binary image of the hand-drawn curve. **b** Binary image of the hand-drawn curve. (c) Skeletonized image of the hand-drawn curve. (d) The image composed of the generated coordinate points.

Figure S6 illustrates the process of recognizing and generating coordinates for hand-drawn curve images. First, the target image is loaded in grayscale mode using the OpenCV library and converted into a binary image by global thresholding (Figure S6 b). Next, the binary image is inverted to swap the foreground and background colors for subsequent skeletonization. Skeletonization is performed using the Zhang-Suen algorithm[1], which progressively decimates the foreground pixels until a slender skeleton is obtained that represents the main structure of the image (Figure S6 c). Then, by recognizing the contours in the skeleton image and selecting the contour with the largest area as the main feature line. Finally, the cumulative distance along this contour is calculated and a specified number of equidistant points are uniformly generated on the contour using linear interpolation. The number of generated coordinate points is 200, and these coordinates are shown as white dots labeled on an image with an all-black background (Figure S6 d).

During the 4D printing process of hydrogels, the hydrogel solution undergoes a light-induced polymerization reaction under UV irradiation, forming a crosslinked hydrogel structure. In this study, we kept the UV light scanning intensity and speed constant while controlling the material properties of

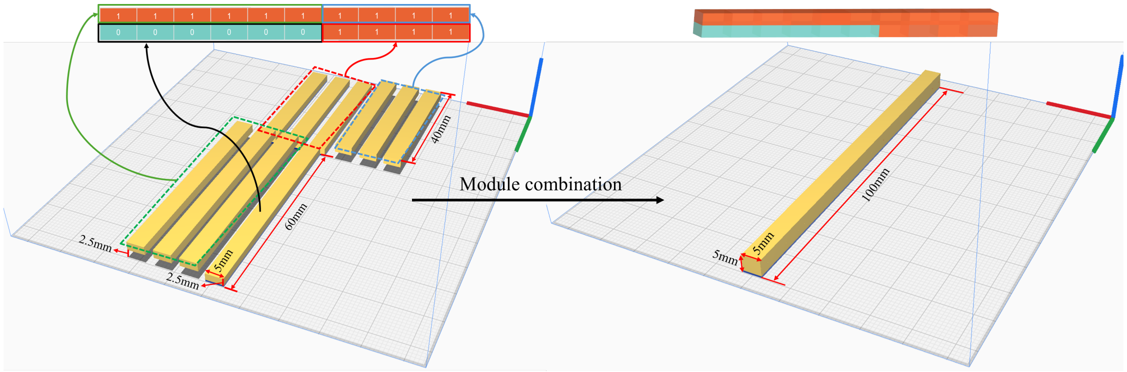


**Figure S7**. **Model construction of 4D-printed hydrogels.**

each voxel unit by changing the number of scans. As shown in Figure S7, in the 3D printing software (UltiMaker Cura), we represented the number of UV light scans by stacking yellow blocks. The code "1" represents three UV light scans, indicated by stacking three yellow blocks, while the code "0" represents one scan, indicated by one yellow block. By stacking these modules, we constructed a beam-shaped structure with dimensions of 100mm in length, 5mm in width, and 5mm in height.

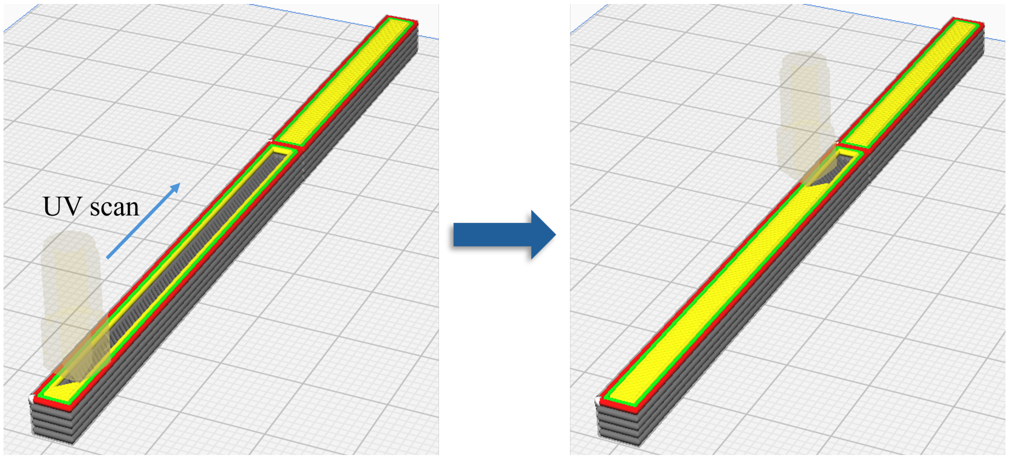


**Figure S8**. **UV light scanning diagram.**

Figure S8 shows the UV light scanning method. First, UV light follows the contours of the model (red and green areas) to print the outer contours, and then the yellow area is scanned layer by layer to complete the overall printing. This printing method can precisely control the material properties of each voxel unit and ensure the uniformity of the material distribution.

The 4D printing equipment used in this study is a first-generation printer developed by Yamagata University. The printer consists of a UV light emitter, a hydrogel container, a printing platform, and a hydrogel solution, as shown in Figure S9. The UV light emitter can move in the X and Y axis directions to scan the hydrogel solution. The printing platform can move up and down along the Z-axis to print the hydrogel layer by layer.

Figure S10 shows examples of hydrogel deformation compared with the FE simulation results. The results indicate that the hydrogel deformation in the actual environment is basically consistent with the simulation results. Under stimulation, the two half-heart-shaped hydrogels successfully combine into a left-right symmetric heart-shaped structure. This demonstrates that our method can accurately control the material properties of each voxel unit, and the hydrogel exhibits a stable stimulus response in real-world environments without significant deviation. In addition, the consistency and repeatability of the experimental results have also been verified, further proving the reliability of the experimental results.

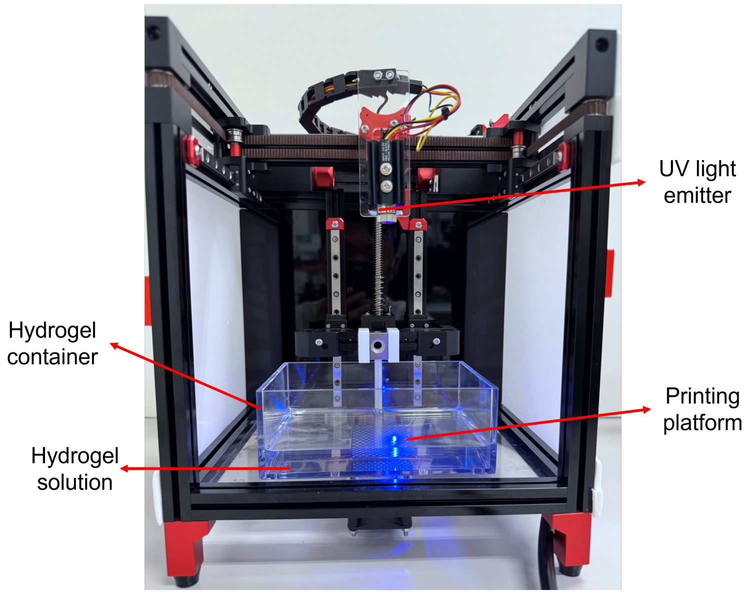


**Figure S9**. **4D printing equipment.**

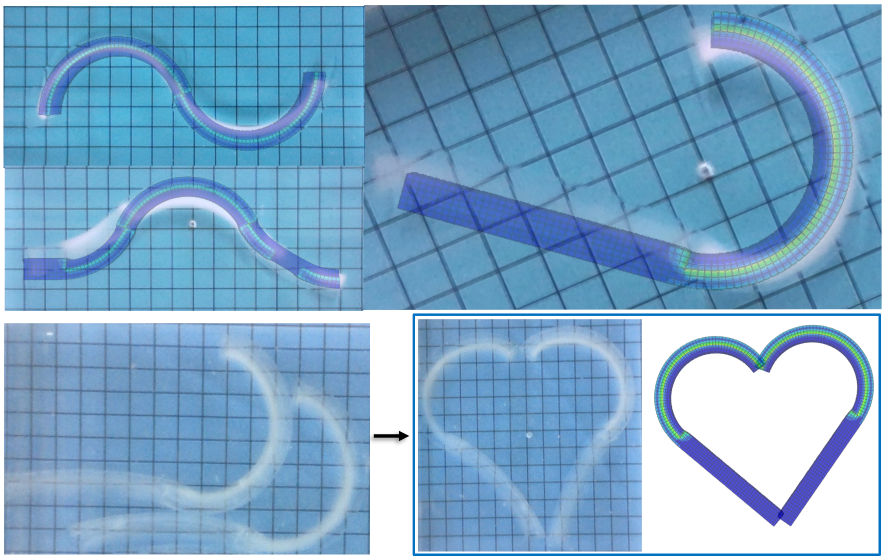

**Figure S10**. **Comparison of hydrogel deformations with FE simulation results.**

# References

[1] T. Y. Zhang, C. Y. Suen, *Commun Acm* **1984**, *27*, 236.
